# Gamma-Ray-Induced Amino Acid Formation in Aqueous Small Bodies in the Early Solar System

**DOI:** 10.1021/acscentsci.2c00588

**Published:** 2022-12-07

**Authors:** Yoko Kebukawa, Shinya Asano, Atsushi Tani, Isao Yoda, Kensei Kobayashi

**Affiliations:** † Department of Chemistry and Life Science, Yokohama National University, 79-5 Tokiwadai, Hodogaya-ku, Yokohama, Kanagawa240-8501, Japan; ‡ Graduate School of Human Development and Environment, Kobe University, 3-11 Tsurukabuto, Nada-ku, Kobe, Hyogo657-8501, Japan; § Co60 irradiation facility, Laboratory for Zero-Carbon Energy, Institute of Innovative Research, Tokyo Institute of Technology, 2-12-1 Ookayama, Meguro-ku, Tokyo, 152-8550, Japan

## Abstract

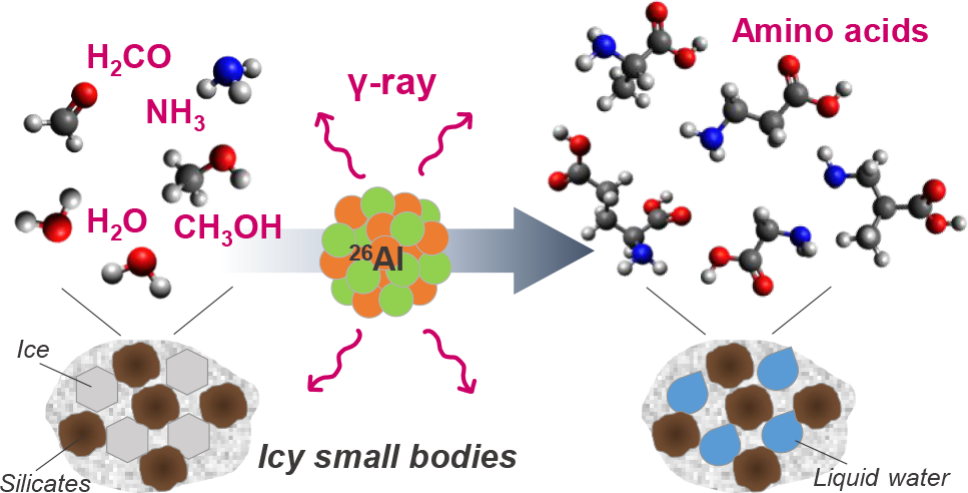

Carbonaceous chondrites contain life’s essential building blocks, including amino acids, and their delivery of organic compounds would have played a key role in life’s emergence on Earth. Aqueous alteration of carbonaceous chondrites is a widespread process induced by the heat produced by radioactive decay of nuclides like ^26^Al. Simple ubiquitous molecules like formaldehyde and ammonia could produce various organic compounds, including amino acids and complex organic macromolecules. However, the effects of radiation on such organic chemistry are unknown. Hence, the effects of gamma rays from radioactive decays on the formation of amino acids in meteorite parent bodies are demonstrated here. We discovered that gamma-ray irradiation of aqueous formaldehyde and ammonia solutions afforded a variety of amino acids. The amino acid yields had a linear relationship with the total gamma-ray dose but were unaffected by the irradiation dose rates. Given the gamma-ray production rates in the meteorite parent bodies, we estimated that the production rates were reasonable compared to amino acid abundances in carbonaceous chondrites. Our findings indicate that gamma rays may contribute to amino acid formation in parent bodies during aqueous alteration. In this paper, we propose a new prebiotic amino acid formation pathway that contributes to life’s origin.

## Abstract 2

We experimentally demonstrate that various amino acids were produced by gamma-ray irradiation to aqueous solutions of NH_3_, H_2_CO, and CH_3_OH simulating aqueous alteration in small bodies in the early solar system.

## Introduction

Amino acids found in carbonaceous chondrites are of interest for cosmochemistry and in the study of the origin of life; however, the origins of these amino acids are not well-known. Icy grains in the interstellar and outer solar nebulae could provide reactive environments for prebiotic chemistry made possible by the energy provided by ultraviolet (UV) light and cosmic rays. The results of laboratory experiments, for example, indicated that interstellar ice analogues irradiated with protons or UV light at ∼10 K and subsequently warmed up to room temperature produced amino acid precursors that were then acid-hydrolyzed to produce amino acids.^1−3^ The parent bodies of chondritic meteorites were formed by the accretion of icy dusts ∼4.5 billion years ago; they then underwent aqueous alteration, as made evident by the presence of hydrated minerals. Furthermore, as a result of aqueous alteration, the complexity and diversity of organic compounds are expected to have increased. Indeed, the results of hydrothermal experiments indicate that amino acids and macromolecular organic matter were produced during aqueous alteration.^4−9^ Notably, ammonia and aldehydes^6,7,9−11^ or hexamethylenetetramine^8^ have been used to successfully synthesize various amino acids. Ammonia and aldehydes produced macromolecular organic compounds similar to those found in carbonaceous chondrites, as well as N-containing cyclic compounds and various other organic compounds.^4,5,12,13^ Radioactive nuclides like ^26^Al have been regarded as the most effective heat sources that drive the aqueous alteration process.^14−16^ The ^26^Al nuclide undergoes conversion to ^26^Mg with β^+^ decay with a half-life of 7.17 × 10^5^ y and a decay constant of 3.01 × 10^–14^ s^–1^. The decay process produces 3.12 MeV per atom mostly as gamma rays.^17^ In the case of the parent body of the most common carbonaceous chondrites—CM (Mighei-group) chondrites, the total gamma-ray radiation produced in the parent body should be 6.3 MGy based on the canonical values for the ^26^Al/^27^Al ratio (∼5 × 10^–5^)^16^ and the abundance of Al in the Murchison meteorite (1.14 wt %).^18^ It should be noted that the canonical ^26^Al/^27^Al ratio highly depends on the timing of asteroid formation—earlier formation leads to higher values. The effects of such radiations on organic matter formation, on the other hand, have not been studied. In the present study, we used gamma-ray irradiation to simulate the aqueous alteration of the meteorite parent body.

## Results and Discussion

### Amino Acid Formation by Gamma-Ray Irradiations

We prepared aqueous solutions of ammonia, formaldehyde, and methanol in sealed glass tubes with the following molar ratios: H_2_O:NH_3_:HCHO:CH_3_OH = 100:6:8:1 (2.8 M ammonia, 3.7 M formaldehyde, and 0.47 M methanol). Although the initial compositions of chondrite parent bodies are unknown, in similar—but not aqueously altered—bodies like comets, simple molecules such as NH_3_, HCHO, and CH_3_OH are expected to be found in the following mutual ratios: H_2_O:NH_3_:HCHO:CH_3_OH = 100:≤1.5:≤4:≤4.^19^ The aforementioned sealed glass tubes containing the described solutions were then irradiated with gamma rays emitted from ^60^Co gamma-ray sources at various dose rates and durations—from 0.5 to 20 kGy h^–1^ and from 3 to 20 h, respectively.

**Figure 1:**
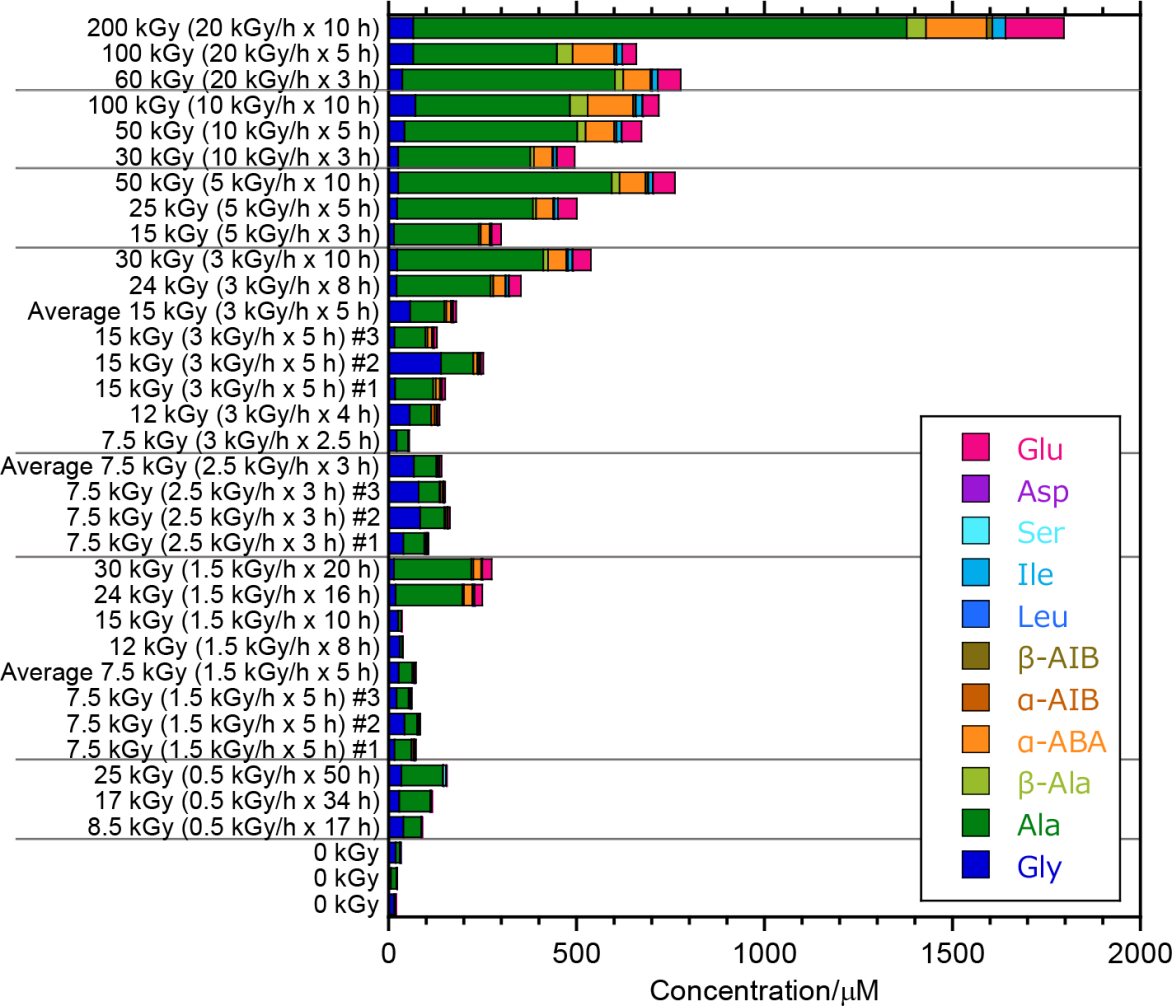
Amino acids produced as a result of gamma-ray irradiation experiments conducted on ammonia, formaldehyde, and methanol aqueous solutions. Notably, “0 kGy” indicates nonirradiated solutions, which were used as controls. The experiments at values for the gamma-ray dose rate and test duration of 3 kG h^–1^ × 5 h, 2.5 kG h^–1^ × 3 h, and 1.5 kG h^–1^ × 5 h were conducted in triplicates; the average and raw results are reported.

**Figure 2:**
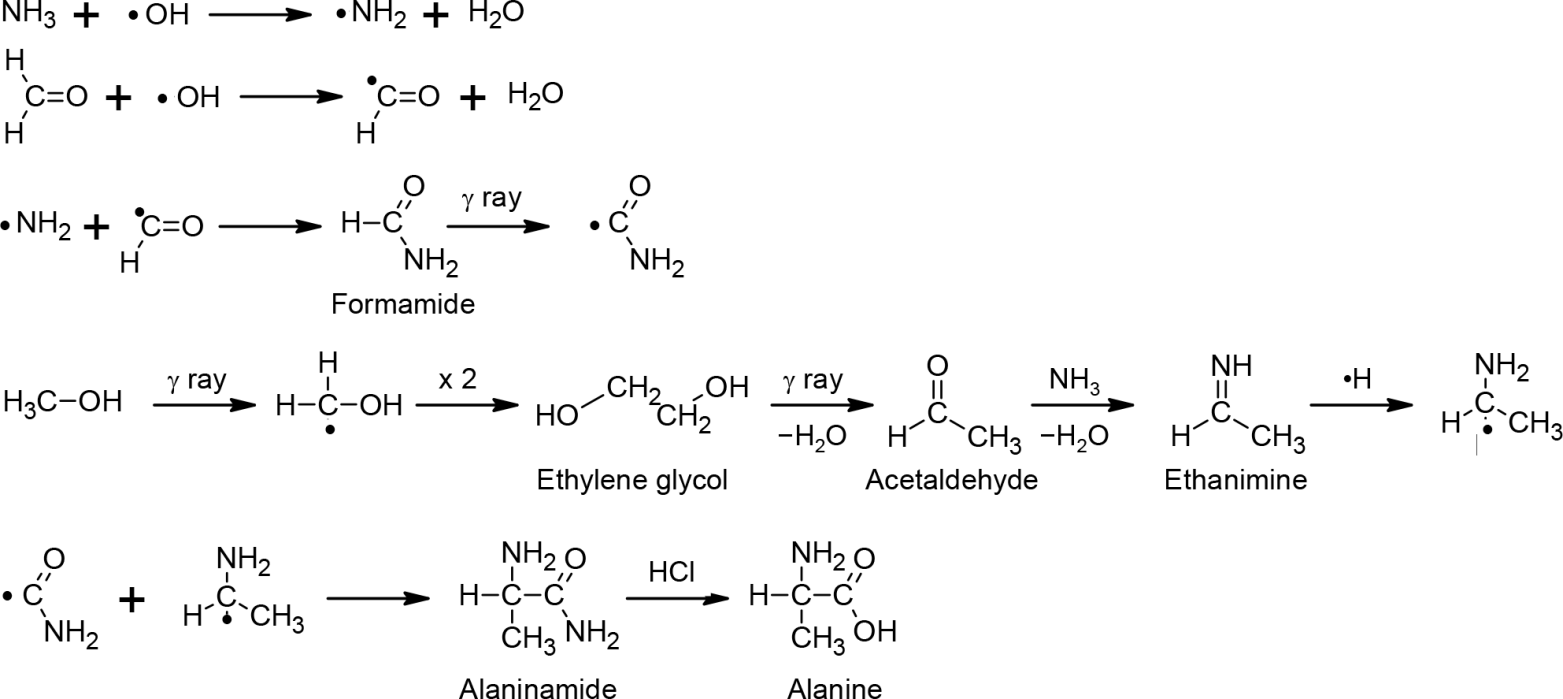
Possible pathways for alanine formation from ammonia, formaldehyde, and methanol aqueous solutions with gamma-ray irradiation based on the radical formations induced by gamma rays^20−26^ in addition to the formation of alanine from ammonia and aldehyde.^7^

Gamma-ray irradiation of formaldehyde and ammonia aqueous solutions (Figures 1 and S1; Table S1) resulted in amino acid production in amounts that were generally superior to those produced in the control samples, which consisted of nonirradiated formaldehyde and ammonia solutions. After acid hydrolysis of the irradiated samples, α-amino acids, mostly alanine with some glycine, as well as α-aminobutyric acid (ABA) and glutamic acid, were found to have formed. β-Amino acids (i.e., β-alanine and β-aminoisobutyric acid (AIB)) were also found to have been produced. Note that these amino acids were attempted to be identified using standards, and thus one cannot exclude the possibilities of formations of other amino acids. These results were somewhat similar to those obtained in heating experiments previously conducted by our group on ammonia, formaldehyde, and methanol aqueous solutions with similar—but slightly different—concentrations,^9^ but alanine was significantly dominant in our gamma-ray irradiation products. High-energy gamma rays are likely to trigger the decomposition of HCHO, NH_3_, CH_3_OH, and H_2_O into radicals. One of the possible reaction pathways could be illustrated as Figure 2, based on the radical formations induced by gamma rays^20−26^ in addition to the formation of alanine from ammonia and aldehyde.^7^ OH radicals produced by gamma-rays efficiently remove hydrogen from molecules and trigger further radical reactions. NH_2_ radical and HCO radical are produced by ammonia^20^ and formaldehyde,^21^ and then formamide is produced.^22^ Ethylene glycol is produced from the CH_2_OH radial which is formed from methanol by gamma rays.^23,24^ Ethylene glycol then produces acetaldehyde^25^ which reacts with ammonia and produces ethanimine.^26^ Ethanimine could react with a hydrogen radical and produce CH_3_CHNH_2_ radical and then react with NH_2_CO radical and produce alaninamide. Finally, acid hydrolysis of alaninamide produces alanine.^7^

We prepared three separate samples for each gamma-ray irradiation experiment conducted at values for the gamma-ray dose rate and test duration of 3 kG h^–1^ × 5 h, 2.5 kG h^–1^ × 3 h, and 1.5 kG h^–1^ × 5 h, and satisfactory reproducibility was observed, except for the case of the amount of glycine detected in one sample (3 kG h^–1^ × 5 h sample #2) (Figure 1 and Table S1). Glycine production was inconsistent in general—likely due to relatively high concentration of glycine in the control samples, so we did not use this amino acid in our quantitative analysis. Furthermore, we confirmed that the d/l ratio of the alanine produced by gamma-ray irradiation was approximately 1, indicating that environmental contamination was minimal (see Supporting Information).

### Correlation between Amino Acid Yields and Gamma-Ray Dose

**Figure 3:**
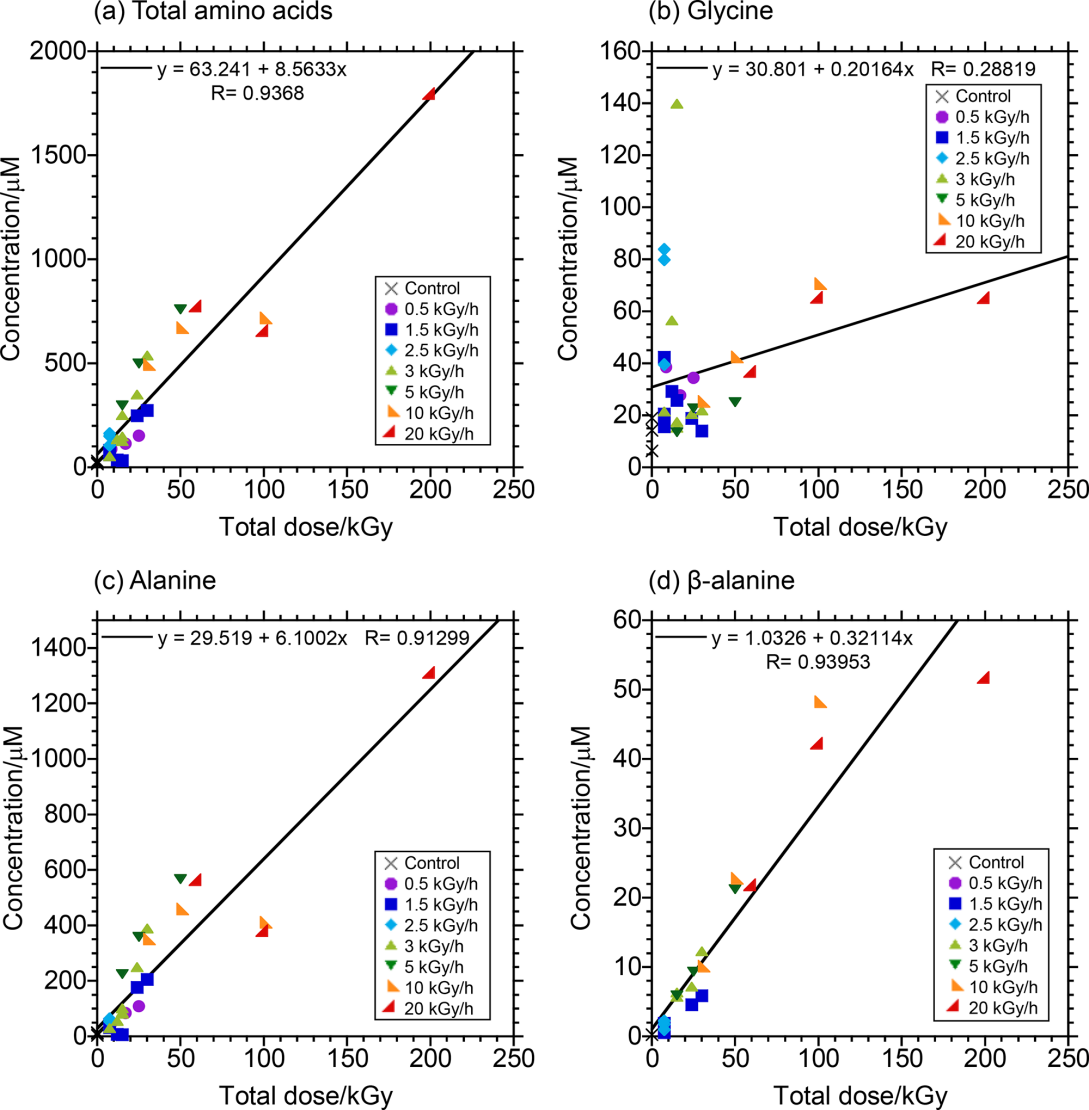
Amino acid yields as a function of the total gamma-ray dose. (a) Total amino acid, (b) glycine, (c) alanine, and (d) β-alanine concentrations versus the total gamma-ray dose.

The amino acid yields increased with the total gamma-ray dose irradiated on the samples (Figure 3a–d). The 200 kGy (20 kGy h^–1^ × 10 h) of irradiation resulted in the highest yield of amino acids—1.797 mM in total which is equivalent to 5.918 mM of carbon. Considering that the starting solution contains 4.22 M of carbon, 0.14% of carbon was converted to amino acids. In fact, the amounts of alanine and β-alanine produced were highly correlated with the total gamma-ray dose (*R* = 0.91 and *R* = 0.94, respectively). No correlation was instead observed between glycine yield and gamma-ray dose. Possibly, as a result of the high proportion of alanine in the total amino acids produced, the total amino acid yield correlated well with the total gamma-ray dose (*R* = 0.94). Indeed, the relationships just described were unaffected by changes in the gamma-ray dose rate (Gy h^–1^). To infer the effects on the amino acid yields of the gamma-ray dose rates, the amino acid yields were plotted against the irradiation time for each value for the gamma-ray dose rate (see Figure S2a–d). In the case of alanine and β-alanine, the amino acid yield and irradiation time correlated fairly well with each other for each dose rate, and the slopes of the plots, which are equivalent to the amino acid yield rates, correlated well with the gamma-ray dose rate (Figure S2e). This confirms the idea that a linear relationship exists between alanine yield and total gamma-ray dose. Specifically, the linear relationship between total gamma-ray dose (*D*) and alanine concentration (*C*_Ala_) could be expressed as follows:
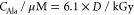
1

The linear relationship between total gamma-ray dose (*D*) and β-alanine concentration (*C*_βala_) could be expressed as follows:
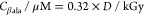
2

These amino acid yields are based on the amino acid concentrations in water. Thus, we estimated the amino acid concentrations in the parent bodies using a water/rock ratio of 0.3–0.6 (v/v) for CM2 chondrites.^27,28^ Notably, the water weight fraction can be calculated as (0.45/2.31)/(1 + 0.45/2.31) = 0.163 using a value for the rock density of 2.31 g cm^–3^ (in the case of the Murchison meteorite^29^) and a value for the water density of approximately 1 g cm^–3^. The amino acid yield per g of meteorite parent body (*m*) can thus be expressed as follows with the molar mass of alanine (89.09 g mol^–1^):
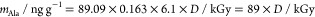
3
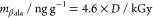
4

### Implications for Amino Acid Formation in Parent Bodies

One can estimate the expected amino acid yields in the parent bodies using the dynamic correlations represented by eqs 1–4. The number of ^26^Al atoms (*N*_26Al_) can be calculated as follows:
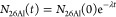
5where λ denotes the decay constant of ^26^Al (λ = 3.01 × 10^–14^ s^–1^). The gamma-ray dose is linearly correlated to the number of decayed ^26^Al [= *N*_26Al_(0) (1– e^–*λt*^)]. Therefore, the total gamma-ray dose *D* and the gamma-ray dose rate d*D*/d*t* can be calculated based on eq 5, as well as on the value of the total gamma-ray dose (6.3 MGy), as follows:
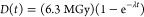
6
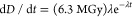
7

**Figure 4:**
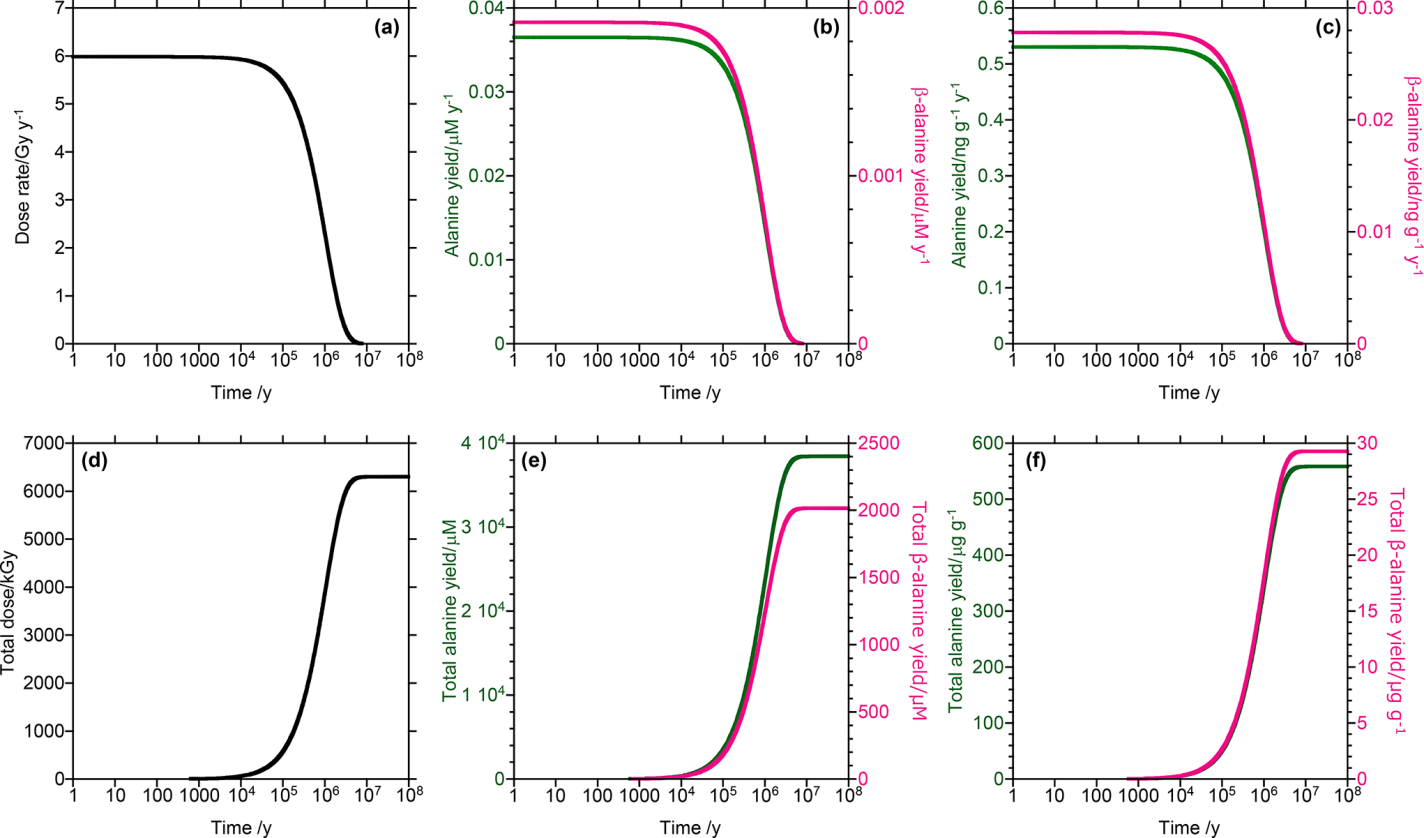
Calculated yields of alanine and β-alanine. (a) Gamma-ray dose rate expected in the parent bodies of CM chondrites, and calculated yields of alanine and β-alanine per year (b) in the liquid phase and (c) in the whole rock. (d) Total gamma-ray dose expected in the parent bodies of CM chondrites and calculated total yields of alanine and β-alanine (e) in the liquid phase and (f) in the whole rock.

The alanine and β-alanine yields were calculated using eqs 1–4; the values thus obtained are presented in Figure 4 and Table 1. Based on the calculated data, a time duration between 10^3^ and 10^5^ y is required for amino acids alanine and β-alanine to be produced in the 1.3–1.4-μg g^–1^ concentration ranges observed in the Murchison meteorite.^30^ These are just rough estimates, however, and not all amino acids found in meteorites can be attributed to gamma-ray-induced reactivity. Nevertheless, evidence from the present study indicates that gamma rays may contribute to the formation of amino acids in meteorites.

**Table 1:** Calculated Yields of Alanine and β-Alanine in the CM Parent Body^a^

		yield rate/μM y^–1^		yield rate/ng g^–1^ y^–1^			total yield/μM		total yield/μg g^–1^	
time/y	dose rate/Gy y^–1^	alanine	β-alanine	alanine	β-alanine	total dose/kGy	alanine	β-alanine	alanine	β-alanine
1	6.0	0.037	0.0019	0.53	0.028	0.0060	0.037	0.0019	0.00053	2.8 × 10^–5^
10	6.0	0.037	0.0019	0.53	0.028	0.060	0.37	0.019	0.0053	0.00028
100	6.0	0.037	0.0019	0.53	0.028	0.60	3.7	0.19	0.053	0.0028
1,000	6.0	0.036	0.0019	0.53	0.028	6.0	36	1.9	0.53	0.028
10,000	5.9	0.036	0.0019	0.53	0.028	60	3.6 × 10^2^	19	5.3	0.28
100,000	5.4	0.033	0.0017	0.48	0.025	5.7 × 10^2^	3.5 × 10^3^	1.8 × 10^2^	51	2.7
1,000,000	2.3	0.014	0.00074	0.21	0.011	3.9 × 10^3^	2.4 × 10^4^	1.2 × 10^3^	3.4 × 10^2^	18
10,000,000	0.00045	2.7 × 10^–6^	1.4 × 10^–7^	4.0 × 10^–5^	2.1 × 10^–6^	6.3 × 10^3^	3.8 × 10^4^	2.0 × 10^3^	5.6 × 10^2^	29

a Amounts of alanine and β-alanine expected to be produced in the parent body of CM chondrites reported in yields per year (yield rates) and total yields in the liquid phase (μM) as well as in the whole rock (ng g^–1^ or μg g^–1^).

Note that the actual gamma-ray source in the parent bodies was mainly ^26^Al, but its half-life is 7.17 × 10^5^ y, which is unrealistic to use for laboratory experiments. Thus, we use ^60^Co (half-life of 5.27 y). In addition to the half-life (expected dose rate), the decay energies of ^60^Co (1.173 MeV and 1.332 MeV) are different from those of ^26^Al, but the difference in decay energies should not significantly affect the number of molecules formed per energy depositions.

Although the apparent amino acid production rate (*k*) obtained here includes the effects of decomposition of the amino acids during the gamma-ray irradiation, the long-term stability of amino acids is worth discussing in the present context. According to the decomposition kinetics data collected in a previously published study, at room temperature, 50% of alanine is expected to decompose in aqueous solution over a period of 1.5 × 10^7^ y; moreover, 99.9% of alanine is expected to decompose over ∼1.5 × 10^8^ y under the same conditions.^31^ The said time frames are long enough for the specified amino acids to have the opportunity to form (<10^7^ y of alteration). However, the half-life of alanine was determined to decrease to ∼10^5^ y at 50 °C and ∼10^3^ y at 80 °C.^31^ As a result, the final alanine concentration in the parent bodies should have been affected by the thermal decomposition of alanine if the alteration temperature was over several tens of degrees Celsius. In general, β-alanine is thermodynamically more stable than alanine; however, both have been found to exhibit similar stabilities under alkaline conditions.^32^ Based on our findings, the very similar abundances of alanine and β-alanine observed in the Murchison meteorite (∼1.3 and ∼1.4 μg g^–1^, respectively)^30^ despite the preferential formation of alanine over β-alanine could be attributed to the higher stability of β-alanine. Notably, the above discussion is relevant to free amino acids; however, amino acids bound to macromolecular species, which would yield amino acids as a result of acid hydrolysis, would exhibit higher stability than free amino acids. For example, amino acids present in the complex amino acid precursor synthesized by proton irradiation of gas mixtures, in a process intended to simulate the interstellar medium, exhibited higher stability than free amino acids when subjected to heating^33^ or gamma-ray irradiation.^34,35^ Solid-state amino acids are known to be gamma-ray-resistant; for example, in the solid state, ∼70% of alanine was observed to survive irradiation with a 3.2-MGy dose of gamma rays,^36^ although decomposition of amino acids was accelerated at least a factor of 10 with the presence of dry silica powder.^37^ Thus, following the aqueous alteration, amino acids should be stable over long periods of time at low temperatures (<0 °C) in the interior of asteroids.

## Conclusions

A Strecker-like reaction involving HCN, ammonia, and aldehydes (or ketones) in the presence of water was the classic scenario for α-amino acid formation in meteorite parent bodies, and a Michael addition of ammonia to unsaturated nitriles was the classic scenario for the formation of β-amino acids in the same context.^38,39^ Moreover, aldehydes and ammonia can produce a variety of amino acids, including α-, β-, and γ-amino acids,^6,7,9,10^ as well as hexamethylenetetramine, which easily decomposes into formaldehyde and ammonia.^8^ In addition to these possibilities, gamma rays could assist the formation of amino acids in the parent bodies. Our findings point to the possibility of gamma-ray-induced amino acid formation from ubiquitous simple molecules such as formaldehyde and ammonia in the presence of water inside small bodies during the early stages of the formation of the Solar System. The gamma-ray-induced production of amino acids could be a novel prebiotic amino acid formation pathway that could have contributed to the origins of life on early Earth, as building blocks of life were delivered through the fall of meteorites.

## Methods

### Gamma-Ray-Irradiation Experiments

The sample solutions were prepared according to the method described by Elmasry et al.^9^ In detail, 200 μL of starting solutions containing 2.8 M ammonia, 3.7 M formaldehyde, and 0.47 M methanol at molar ratios of H_2_O:NH_3_:HCHO:CH_3_OH = 100:6:8:1 was prepared from 55.5 μL of 37% (mass/mass) formaldehyde aqueous solution (containing 5% methanol) and 41.5 μL of 25% ammonia aqueous solution purchased from Wako Pure Chemical Corporation, in addition to 103 μL of pure water. After O_2_ was removed from the system by keeping it under a vacuum while cooling it with liquid nitrogen, each solution was flame-sealed into glass tubes (6 mm in diameter). Even though we attempted to remove O_2_, some O_2_ could be left in the sample tubes, but O_2_ in general tends to inhibit the formation of amino acids by oxidation, and thus at least, we did not overestimate the yields of amino acids. These solution-containing tubes were exposed to gamma-ray irradiation at ambient temperature using ^60^Co sources at the Laboratory for Nuclear Reactors, Tokyo Institute of Technology and the Research Laboratory for Quantum Beam Science, Institute of Scientific and Industrial Research, Osaka University (Rabbit11) (Figure S3). The same protocols were employed to prepare the control samples, which were not irradiated with gamma rays. All the utilized glassware was baked at 500 °C for 4 h prior to use in all experiments, and the water used in the experiments and analytical procedures was prepared using a Millipore Milli-Q system.

Note that the temperatures during gamma-ray irradiation experiments should not exceed ∼70 °C, even if all the energy was used to increase temperature for the 200 kGy (maximum dose) experiment. Comparing to the heating experiment without gamma rays at 80 °C for 24 h (Table S1), the total amino acid yield was ∼15 μM, which is much less compared to those of ∼1800 μM produced by 200 kGy of gamma-ray irradiation. This fact excludes the possibility of the effects of heating by gamma-ray irradiation in our experiments.

### Amino Acid Analyses

The amino acid analyses were conducted according to the method described by Kebukawa et al.^6^ The gamma-ray-irradiated and control samples were acid-hydrolyzed with 6 M HCl for 24 h at 110 °C to release the bound amino acids, which is the standard technique for amino acid analysis.^40,41^ Following acid hydrolysis, the samples were dried by vacuum centrifugation conducted at 60 °C. Each dried sample was then dissolved in 0.4 mL up to 1.5 mL of water (depending on the amino acid concentrations). A 10-μL aliquot of each of the obtained aqueous sample solutions was filtered through a 0.45-μm membrane filter before being analyzed using a high-performance liquid chromatography (HPLC) system. Notably, the HPLC system included a system controller (Shimadzu CBM-20A), HPLC pumps (Shimadzu LC-20AD), a polystyrene-type ion exchange column (Shimadzu Shimpack ISC-07/S1504) heated at 55 °C in a column heater (CTO-20AC), and a fluorescence detector (Shimadzu RF-20Axs) with a 358 nm excitation wavelength and a 450 nm emission wavelength. A postcolumn derivatization process was conducted using a solution comprising *o*-phthalaldehyde 0.104 g L^–1^, *N*-acetyl-l-cysteine 0.65 g L^–1^, Na_2_CO_3_ 40.7 g L^–1^, H_3_BO_3_ 13.5 g L^–1^, K_2_SO_4_ 18.8 g L^–1^, and polyoxyethylene lauryl ether 0.2 g L^–1^. Gradient elution was performed using a Shimadzu amino acid mobile phase kit (Na type) with the following eluents: (A) pH 3.23 sodium citrate buffer (containing 0.2 M Na^+^ and 7% (v/v) ethanol); (B) pH 10.00 sodium citrate buffer (containing ca. 0.73 M Na^+^ and 0.2 M boric acid); and (C) 0.2 M sodium hydroxide aqueous solution (for conditioning purposes). The flow rate of the carrier solution was 0.300 mL min^–1^ with (A) and (B) gradients; 100% (A) at first 0–15 min, the ratio of (B) was increased from 0% to 16% at 15–35 min, 16% (B) was kept at 35–40 min, the ratio of (B) was increased from 60% to 100% at 40–50 min, 100% (B) at 50–60 min, and finally 100% (A) at 60–65 min. Aspartic acid, serine, glutamic acid, glycine, alanine, α-aminoisobutyric acid, α-aminobutyric acid, isoleucine, leucine, β-alanine, and β-aminoisobutyric acid were quantified using commercial amino acid standard solutions (Wako Amino Acids Mixture Standard Solution, Type B and Type AN-2), in addition to an α-aminoisobutyric acid standard.

### Gas Chromatography/Mass Spectrometry Analysis of Alanine

We also conducted gas chromatography/mass spectrometry (GC/MS) measurements to determine the d/l ratio of alanine to ensure that the obtained amino acids were not contaminated by environmental sources, based on the fact that environmental alanine is characterized by a low d/l ratio, whereas the gamma-ray-triggered alanine synthesis would produce a racemic mixture for the said amino acid. Prior to GC/MS analysis, the acid-hydrolyzed samples were derivatized using the method described by Ubukata et al. (2007).^42^ Subsequently, 45 μL of 2,2,3,3,4,4,4-heptafluoro-1-butanol and 15 μL of pyridine were combined in a small vial and vortexed for ∼10 s. The sample solution (50–100 μL) was transferred into the vial, which was then vortexed. Afterward, 30 μL of chloroform and 10 mg of NaCl were added to the vial, before the said vial was vortexed once again. A syringe was used to inject 1 μL of the organic phase present in the vial into a GC/MS system (Shimadzu QP-2020) with an Agilent J&W CP-Chirasil-l-Val capillary column (25 m × 0.25 mm i.d. and 0.12-μm film thickness). A splitless injection mode was used; an injector temperature of 200 °C, an inlet pressure of 40.6 kPa, and a He flow of 1 mL/min were applied. The column temperature was maintained at 50 °C for 1 min, before it was initially made to increase to 150 °C at a rate of 5 °C min^–1^ and then to 200 °C at a rate of 7 °C min^–1^. Electron ionization was conducted at 70 eV, the ion source temperature was set to 200 °C, and scanning was conducted in selected ion monitoring (SIM) mode at *m*/*z* = 116 (the most intense fragmentation peak due to derivatized alanine).

The area ratios of the peaks due to d-alanine and l-alanine in the chromatograms obtained for the gamma-ray-irradiated sample (5 kGy h^–1^ × 3 h) and for the dl-alanine standard had values of 0.840 and 0.877, respectively (Figure S4). Therefore, the value of the d/l-alanine ratio in the gamma-ray-irradiated sample was determined to be ∼0.96, pointing to the racemic nature of the amino acid produced as a result of the gamma-ray-irradiation experiment. Evidence thus indicates that only minor environmental alanine contamination of the samples subjected to irradiation with gamma rays had occurred.

## Supporting Information Available

Supplementary Figure 1, typical HPLC chromatograms of gamma-ray irradiated samples; Figure 2 amino acid yields as a function of irradiation time for each gamma-ray dose rate; Figure 3, photos of the ^60^Co facility and samples before and after the irradiation; Figure 4, GC/MS chromatograms of gamma-ray irradiated sample. Supplementary Table 1, yields of amino acids by gamma-ray irradiations (PDF)
