# Supplementary material for: Gamma-Ray-Induced Amino Acid Formation in Aqueous Small Bodies in the Early Solar System
Source: ACS Cent Sci. 2022 Dec 7;8(12):1664–71. doi: 10.1021/acscentsci.2c00588 (PMC9801502; doi:10.1021/acscentsci.2c00588)
Supplement: Supplementary file 1 — oc2c00588_si_001.pdf [file oc2c00588_si_001.pdf]

Supporting Information

Gamma-ray-induced amino acid formation in aqueous small bodies in the early solar system

Yoko Kebukawa[1]*, Shinya Asano[1], Atsushi Tani[2], Isao Yoda[3], and Kensei Kobayashi[1]

[1]Department of Chemistry and Life Science, Yokohama National University, 79-5 Tokiwadai, Hodogaya-ku, Yokohama, Kanagawa, 240-8501, Japan
[2]Graduate School of Human Development and Environment, Kobe University, 3-11 Tsurukabuto, Nada-ku, Kobe, Hyogo, 657-8501, Japan
[3]Co60 irradiation facility, Laboratory for Zero-Carbon Energy, Institute of Innovative Research, Tokyo Institute of Technology, 2-12-1 Ookayama, Meguro-ku, Tokyo, 152-8550, Japan

*Corresponding author email: kebukawa@ynu.ac.jp

Supplementary figures and table

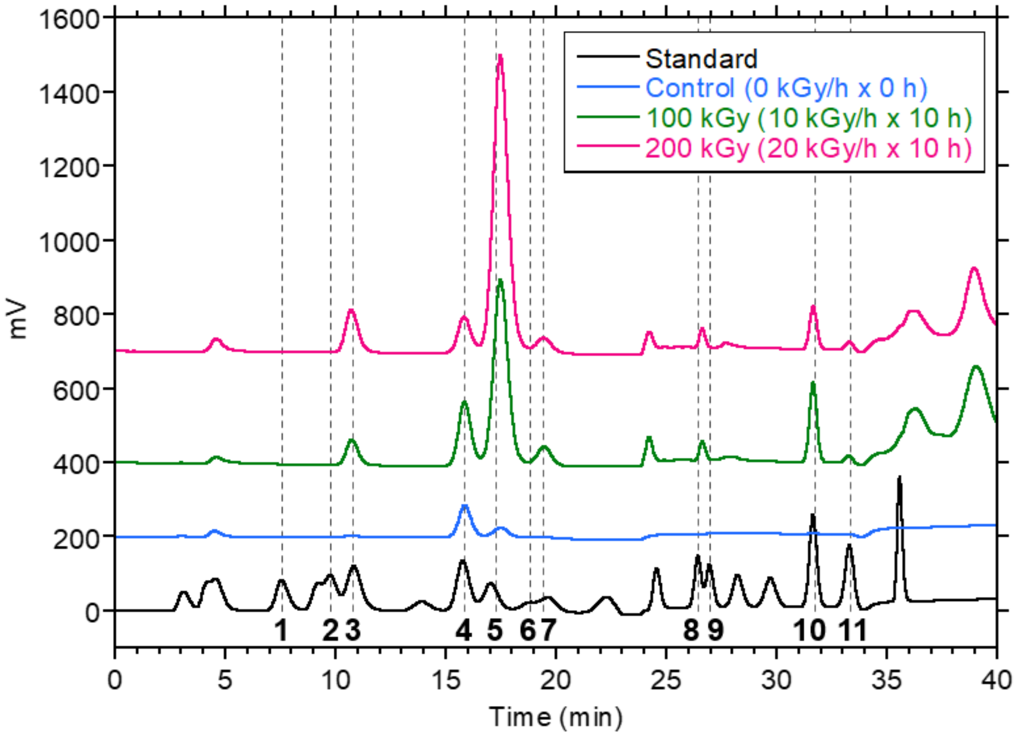

Supplementary figure 1: Typical HPLC chromatograms of gamma-ray irradiated samples (10 kGy h^{-1} × 10 h and 20 kGy h^{-1} × 10 h), control, and amino acid standard. 1: Aspartic acid, 2: Serine, 3: Glutamic acid, 4: Glycine, 5: Alanine, 6: α-aminoisobutyric acid, 7: α-aminobutyric acid, 8: Isoleucine, 9: Leucine, 10: β-alanine, 11: β-aminoisobutyric acid.

Supplementary table 1: Yields of amino acids by gamma-ray irradiations to the aqueous solutions of formaldehyde and ammonia.

Amino acid concentrations in μM	Glycine	Alanine	β-alanine	α-aminobutyric acid	α-aminoisobutyric acid	β-aminoisobutyric acid	Leucine	Isoleucine	Serine	Aspartic acid	Glutamic acid	Total
0 kGy (Control)	14.3	3.6										18.0
0 kGy (Control)	6.4	14.6									1.4	22.4
0 kGy (Control)	19.0	11.4	0.3								1.0	31.7
8.5 kGy (0.5 kGy h^{-1} × 17 h)	38.7	49.5										88.1
17 kGy (0.5 kGy h^{-1} × 34 h)	27.8	84.2							2.5			114.5
25 kGy (0.5 kGy h^{-1} × 50 h)	34.4	110.2							9.0			153.6
7.5 kGy (1.5 kGy h^{-1} × 5 h) #1	15.6	43.9	2.0	4.9		0.3	1.1	1.0		0.3	3.5	72.5
7.5 kGy (1.5 kGy h^{-1} × 5 h) #2	42.4	33.5	0.6	3.3			1.3				2.5	83.6
7.5 kGy (1.5 kGy h^{-1} × 5 h) #3	20.6	32.5	1.2	3.1			0.7				2.6	60.7
Average 7.5 kGy (1.5 kGy h^{-1} × 5 h)	26.2	36.6	1.2	3.8		tr.	1.1	tr.		tr.	2.9	72.3
Standard deviation	14.2	6.3	0.7	1.0			0.3				0.5	11.5
12 kGy (1.5 kGy h^{-1} × 8 h)	29.4	7.3									0.6	37.2
15 kGy (1.5 kGy h^{-1} × 10 h)	25.9	7.0	tr.								0.7	33.6
24 kGy (1.5 kGy h^{-1} × 16 h)	18.9	177.6	4.6	21.8				4.4			21.9	249.1
30 kGy (1.5 kGy h^{-1} × 20 h)	14.0	205.9	5.9	20.6		2.1		tr.			25.8	274.3
7.5 kGy (2.5 kGy h^{-1} × 3 h) #1	39.6	55.8	1.0	4.2			1.9	0.3			3.9	106.7
7.5 kGy (2.5 kGy h^{-1} × 3 h) #2	83.9	63.8	1.8	6.3			2.0				5.8	163.5
7.5 kGy (2.5 kGy h^{-1} × 3 h) #3	79.9	55.2	2.3	6.9			1.6				4.9	150.9
Average 7.5 kGy (2.5 kGy h^{-1} × 3 h)	67.8	58.2	1.7	5.8			1.8	tr.			4.8	140.3
Standard deviation	24.5	4.8	0.6	1.4			0.2				0.9	29.8
7.5 kGy (3 kGy h^{-1} × 2.5 h)	21.6	30.0	tr.						tr.		2.7	54.2
12 kGy (3 kGy h^{-1} × 4 h)	56.6	56.7	tr.		9.1	7.2					5.5	135.1
15 kGy (3 kGy h^{-1} × 5 h) #1	17.5	101.3	6.3	11.6		0.9	3.2	0.8			8.4	150.0
15 kGy (3 kGy h^{-1} × 5 h) #2	139.8	85.9		10.5			4.7	3.3			7.1	251.3
15 kGy (3 kGy h^{-1} × 5 h) #3	15.2	82.2	5.8	12.5		0.2	3.3				8.0	127.3
Average 15 kGy (3 kGy h^{-1} × 5 h)	57.5	89.8	6.0	11.5		0.6	3.7	2.0			7.8	176.2
Standard deviation	71.3	10.1		1.0			0.9				0.7	66.0
24 kGy (3 kGy h^{-1} × 8 h)	20.6	250.8	7.2	30.9		2.8		7.0			32.0	351.2
30 kGy (3 kGy h^{-1} × 10 h)	22.0	390.1	12.3	48.2		4.0		11.8			49.4	537.8
15 kGy (5 kGy h^{-1} × 3 h)	13.6	225.0	5.9	24.0		2.0		3.6			24.3	298.5

25 kGy (5 kGy h^{-1} × 5 h)	22.8	359.9	9.4	45.1	3.2	9.6	49.6	499.6
50 kGy (5 kGy h^{-1} × 10 h)	25.1	568.1	21.3	69.8	6.6	11.7	58.1	760.7
30 kGy (10 kGy h^{-1} × 3 h)	25.5	351.3	10.2	47.8	3.0	9.6	47.4	494.7
50 kGy (10 kGy h^{-1} × 5 h)	42.5	459.2	22.8	76.1	5.0	13.9	53.4	673.0
100 kGy (10 kGy h^{-1} × 10 h)	70.7	411.2	48.3	120.2	6.9	17.7	43.0	718.0
60 kGy (20 kGy h^{-1} × 3 h)	37.0	564.8	21.9	72.3	5.1	13.9	61.8	776.8
100 kGy (20 kGy h^{-1} × 5 h)	65.5	381.6	42.3	110.5	6.2	15.3	37.8	659.1
200 kGy (20 kGy h^{-1} × 10 h)	65.3	1313.2	51.8	160.8	15.1	35.0	155.8	1797
80 °C 24 h (without gamma-ray)	14.6	0.9						15.5

tr.: Trace amount were detected but not suitable for quantification.

Blank: not detected.

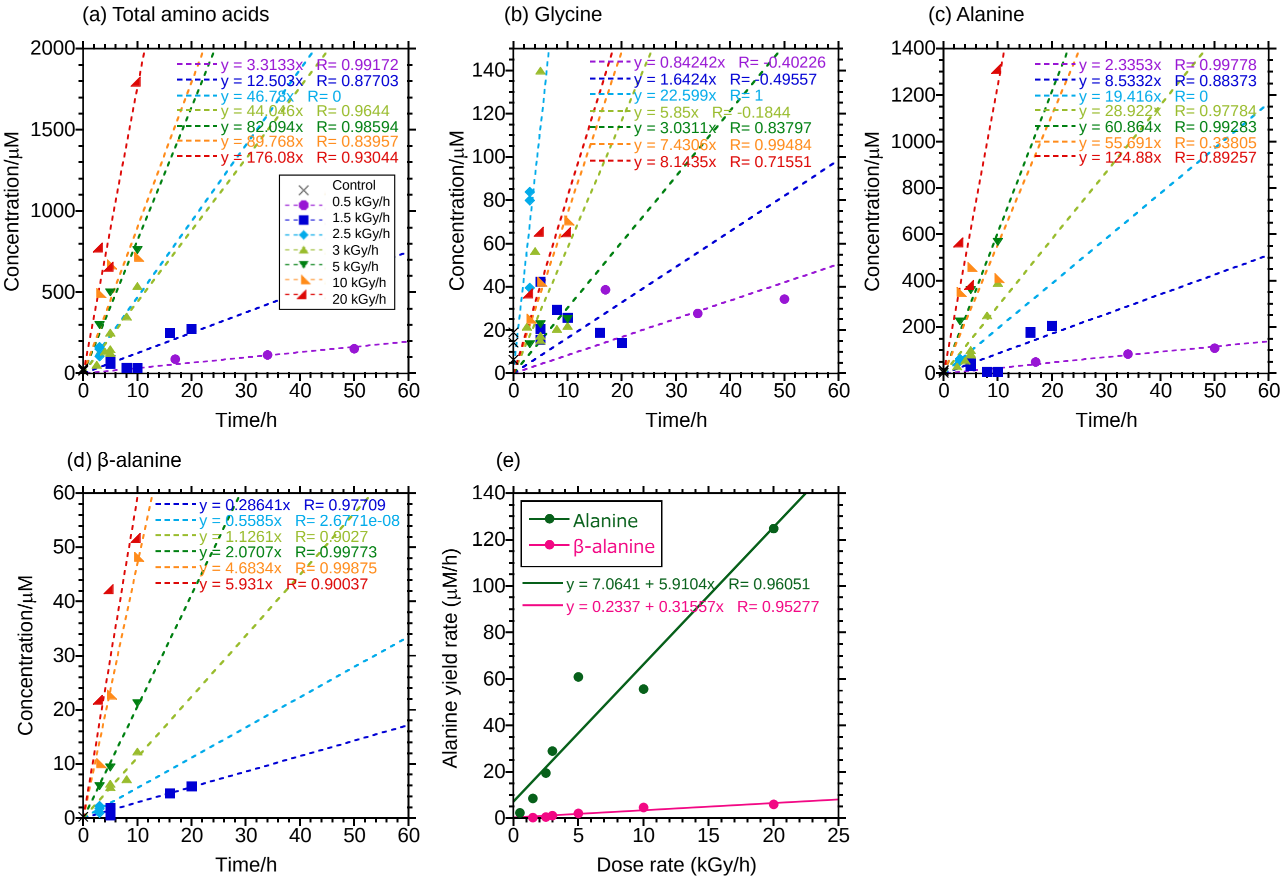

Supplementary figure 2: (a) Total amino acid yield as a function of irradiation time for each gamma-ray dose rate. (b) Glycine yield as a function of irradiation time for each gamma-ray dose rate. (c) Alanine yield as a function of irradiation time for each gamma-ray dose rate. (d) β-alanine yield as a function of irradiation time for each gamma-ray dose rate. (e) Plot of the alanine and β-alanine yield rates as functions of gamma-ray dose rate obtained from the linear approximations reported in (c, d).

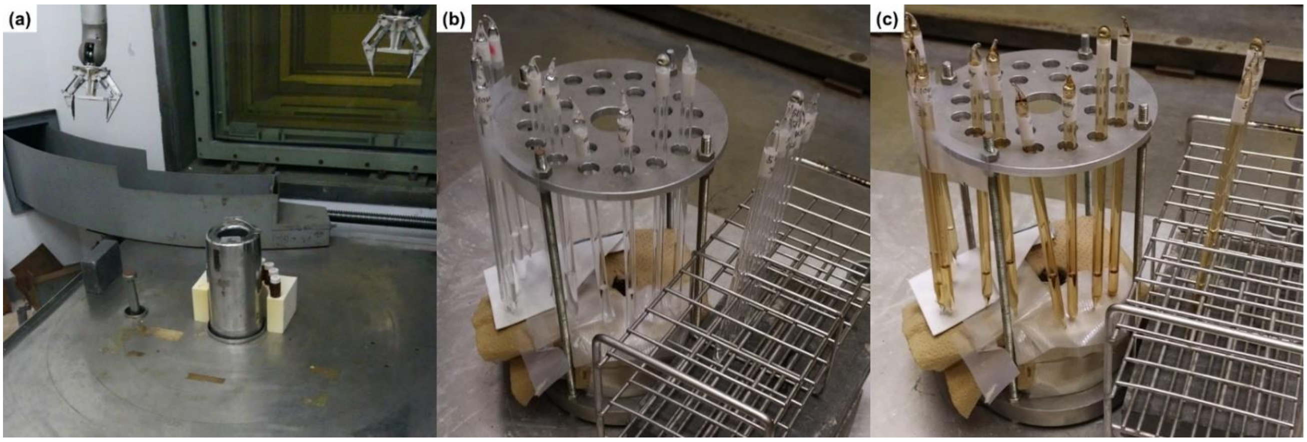

Supplementary figure 3: (a) The ^{60}Co facility at Tokyo Institute of Technology. (b,c) Sample solutions in glass tubes (b) before and (c) after the gamma-ray irradiation at the ^{60}Co facility (Rabbit11) in the Institute of Scientific and Industrial Research, Osaka University. The samples turned brown by gamma-ray.

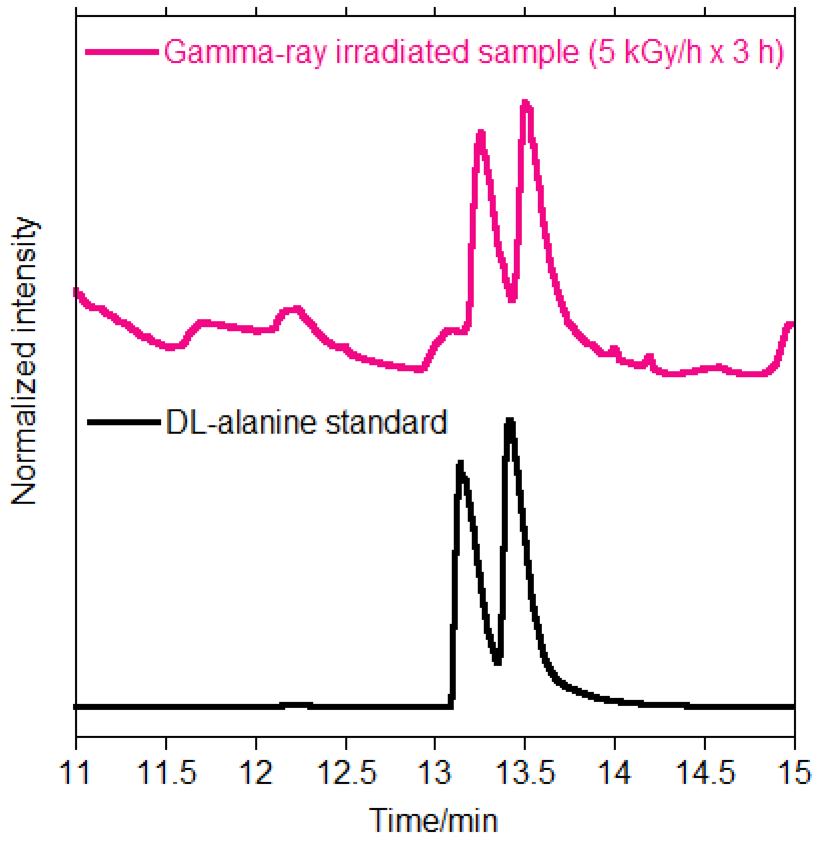

Supplementary figure 4: GC/MS chromatograms of gamma-ray irradiated sample (5 kGy h^{-1} × 3 h) and DL-alanine standard at m/z = 116.
